# Magnitude and effects of food cravings on nutritional status of pregnant women in Southern Ethiopia: A community-based cross sectional study

**DOI:** 10.1371/journal.pone.0276079

**Published:** 2022-10-13

**Authors:** Anteneh Fikrie, Abel Yalew, Anchamo Anato, Wondwosen Teklesilasie

**Affiliations:** 1 School of Public Health, Institute of Health, Bule Hora University, Bule Hora, Ethiopia; 2 Medical Director, Abel Medium Clinic, Gondar Town, Northern Ethiopia; 3 School of Nutrition, Food Science and Technology, College of Agriculture, Hawassa University, Hawassa, Ethiopia; 4 School of Public Health, College of Medicine and Health Sciences, Hawassa University, Hawassa, Southern Ethiopia; University of the Witwatersrand Faculty of Health Sciences / Pan African University, SOUTH AFRICA

## Abstract

**Background:**

Food cravings is a strong and intense urge to consume a specific food and reported as being associated with overweight and overall caloric intake in pregnant women. However, the nutritional and anthropometric consequences are not well recognized. Therefore, this study aimed to assess magnitude and effects of food cravings on nutritional status of pregnant women in Southern Ethiopia.

**Methods:**

A community-based cross-sectional study conducted among 586 randomly selected pregnant mothers at Sidama Regional State from June 1–20, 2019. Pre-tested and semistructured face-to-face interview questionnaires used to collect the data. The data were cleaned, coded, and entered into Epi Data version 3.1 and analysed using SPSS IBM version 20. The bi-variable and multivariable logistic regression used to identify the possible factors of food cravings. Principal component analysis used to determine the wealth status of the study participants. Adjusted Odds Ratio (AOR) together with 95% Confidence Intervals (CIs) used to declare statistical significance.

**Results:**

The study found that nearly three in five, 309 (58.3%) [95%, CI: 54.2%-62.8%] of the study participants reported food cravings. Meat (71.5%) and Fruits (41.7%) were the most frequently craved. About one-third, 194 (36.6%) of the study participants were undernourished (MUAC < 23 cm). Age of women (20–34 years), government employed, Antenatal Care (ANC), Pica practice, lowest wealth quintile, and skipping meals were statistically associated with food cravings. Whereas, wealth quintile and ability to consume craved food were factors associated with the nutritional status of pregnant women. Moreover, our study result found that maternal undernutrition and food cravings were statistically associated (p<0.001).

**Conclusion:**

The prevalence of food cravings in this study is comparable to the global level. However, the magnitude of undernutrition found to be higher. Thus, health care providers need to take every opportunity to encourage women to adopt healthful dietary practices during pregnancy.

## Introduction

Pregnancy is sensitive time of human development, and anything that compromises the fetal environment may have important and lasting effects on the child’s future health [1, 2]. There are innumerable helpful physiological and behavioral changes occurred during pregnancy to sustain and accommodate the new life growing inside her [1]. The physiological changes occur in pregnancy to nurture the developing fetus and prepare the mother for labor and delivery. However, some of these changes influence normal biochemical values while others may mimic symptoms of medical disease [2].

Food cravings, which typically occurred by the end of the first trimester, peak and intensify during the second trimester, and then typically begin to subside are strong and sudden urges to eat particular food while a food aversion is a sense of repulsion at the very thought of a certain kind of food [3]. Food cravings and aversion can serve as a motivator for increasing and/or decreasing the intake of certain food. However, food cravings have been reported as being associated with overweight [4] and are generally reported for foods that provide energy, whereas aversions are more often associated with a response to nausea and vomiting and are therefore likely to be associated with the avoidance of foods and restriction [5]. No evidence exists to suggest that food cravings and aversions are associated with each other [5, 6].

Pregnancy requires a healthy diet that embraces an adequate intake of energy, protein, vitamins and minerals to meet maternal and fetal needs. However, for many pregnant women, dietary intake of vegetables, meat, dairy products, and fruit is often insufficient to meet these needs particularly in low and middle-income countries (LMICs) where multiple nutritional deficiencies often co-exist [7]. On the other hand, food cravings that occurred during the pregnancy motivate the pregnant women to have a desire for sweets, desserts, and chocolates and in a general increase in consumption of sugary foods and beverages and overall caloric intake in pregnant women. This statement has been supported by a study conducted to assess the anthropometric and nutritional associations of food cravings during pregnancy that, those women who craved foods had a higher mean energy intake and gained more weight throughout pregnancy than those who did not crave foods [4].

Several studies reported the magnitude of food cravings from 60 to 85% among pregnant women [3, 5, 6, 8, 9], where the lowest figure comes from Europe, while the highest prevalence corresponds to the African continent [3, 8]. Similarly in Ethiopia, the prevalence of food craving was reported by two different studies revealed in between 43% to 72% among pregnant women [6, 9]. Foods craved for by pregnant women in Western cultures are chocolate, fruits and fruit juices, sweet foods such as ice cream and desserts, and to a lesser extent different types of meat and dairy products [3]. On the other hand, pregnant women in low-income countries craves mainly livestock origin such as meat, yogurt, cheese, and milk followed to a lesser extent by vegetables, fruits, and grains [3, 6, 8, 9]. Dietary cravings alter food intake however the nutritional and anthropometric consequences are unknown [4]. Although food cravings are associated with food intake, not all food cravings result in a nutritious diet. Pica, the craving for non-food substances such as detergent or soil, potentially affects the health of both the mother and fetus [3, 10]. A recent explanation suggests a possible association between cravings and the risk of excessive weight gain during pregnancy and also associated with an increased risk of abnormal glucose tolerance and the development of gestational diabetes mellitus [3]. It has also been linked to obesity, overweight, eating disorders and, given the rise in prevalence of excess gestational weight gain [11]. Hence, food craving can be a potentially modifiable determinant for energy intake (EI) and nutrient quality in pregnancy [3, 4]. Although the specific cause of food cravings during pregnancy is not known, researchers suggest that the hormonal changes during pregnancy have played pivotal role in the development of cravings [4, 10]. Anecdotal evidence suggested that an increased requirement for energy or other nutrients may result in physiological changes in taste and olfactory sensitivity, which can cause cravings [4]. A study reported that the availability of foods has been found to influence the development of cravings [9].

Good maternal nutrition is important for the health and reproductive performance of women and the health, survival, and development of the fetus [5]. Dietary behavior change, a change in the amount and/or choice of food consumed, during pregnancy can affect the amount of energy and nutrients available for fetal growth. Studies showed that food cravings could promote intake of essential nutrient during pregnancy [3, 5]. Likewise, evidence showed that women who craved foods had a higher mean daily energy intake and gained more weight throughout pregnancy than those who did not experience food cravings [4]. Contrary to this, studies conducted in Southern Ethiopia found that food craving was not significantly associated with the nutritional status of pregnant women [6, 9]. This study aimed to assess the magnitude of foods craving, types of food craved, and averted and factors affecting food cravings, and to measure the effect of food cravings on the nutritional status of pregnant women during pregnancy in Sidama Regional State, Southern Ethiopia, 2021.

## Materials and methods

### Study setting, design, and period

The study was conducted at Sidama Regional State, a newly emerged region of the country, Boricha District. The district is one of the 36 administrative districts found in the region. It is located around 32 km away from Hawassa city, the regional legislative city, and 297 km away from Addis Ababa, the capital City of Ethiopia in the southwest direction. The district administratively divided into one urban and thirteen rural kebeles. The district total population is estimated to be 121,648. Of these, 50.5% (61,432) are females and the estimated numbers of pregnant women were 4209. A community-based cross-sectional study conducted from June 1–20, 2019.

### Source, study population, and eligibility criteria

All pregnant women who lived in Sidama region, Boricha district were our source populations whereas pregnant women who lived in a randomly selected five kebeles were our study populations. Pregnant women who were healthy, and had a gestational age of 20 weeks and above were included in this study. On the other hand, pregnant women who lived less than six months in the district and who had chronic diseases were excluded from the study.

### Sample size determination, sampling technique, and procedure

The sample size had calculated by using the single population proportion formula. Considering, the following assumptions: Proportion of food cravings, 59% [10], 5% marginal error (d), 95% confidence level, 1.5 Design effect and 5% of non-response rate. Thus, the sample size calculated became 586. A multistage sampling technique employed to select study participants. At the very beginning, we stratified the total kebeles found in the district into urban and rural. Then only one urban kebele and four rural kebeles selected randomly by using the lottery method. All eligible study subjects identified and registered according to the inclusion criteria house to house by the data collectors. Then a sampling frame, which contains a list of 2216 pregnant women, was prepared. Then after, a systematic sampling technique used to select the actual participants of the study using the k^th^ interval. The (K^th^) interval calculated as K = total population (N)/sample size (n): 2216/586 = 3.7~ 4. Thus, using a lottery method, number 2 selected as the beginning point. Finally, the actual data collected from every fourth pregnant starting from the second pregnant woman.

### Data collection procedures and quality

Primarily the data collection tools were adapted from previous similar studies [8, 9] (S1 File). In addition to this, the adapted questionnaires sent to the nutritionist for further validation. Experienced five first-degree holder in health and two MSc holders in Public Health professionals to the task recruited as data collectors and supervisors respectively. Following the recruitment process, two-days training was conducted for both data collectors and supervisors on research ethical principles, quality data collection techniques, and the basic technique of Mid Upper Arm Circumferance (MUAC) measurement through both demonstration and re-demonstration methods. Furthermore, the investigators also assessed the quality of the data during the data entry and analysis stage to verify the completeness of the collected data. Socio-demographic and economic, maternal meal patterns, and health information-related variables were collected by a pretested and semi-structured face-to-face-interviewer administered questionnaire. Regarding food cravings the study participants were inquired to report if they had incidents of sudden urge for a particular food during their current pregnancy. Further, the women were also asked to list food types which they craved for it. On the other hand, the nutritional status of pregnant women was assessed by using MUAC measuring tape. MUAC was measured at the midpoint between the olecranon and acromion processes using a non-stretchable tape calibrated to 0.1cm [7, 12].

### Study variables and operational definition

#### Nutritional status

Measured by using MUAC and categorized based on the sphere project minimum standards the cut-off point for MUAC measurement. Accordingly, a pregnant woman having MUAC<23.0cm was categorized as undernourished; whereas pregnant women with MUAC ≥23.0cm were categorized as well-nourished [12].

#### Food craving

It is an intense urge to consume a specific food during pregnancy [3].

#### Food aversion

A strong dislike for certain food [3].

### Data processing and analysis

The collected data were check for completeness and consistency, then entered into Epi data version 3.1 and exported to SPSS version 21 for further analysis. Continous variable summarized using the median (±IQR) while categorial variables were describing using frequency and percentages. Likewise, the data presented using statistical tables. Principal component analysis (PCA) was computed for constructing the wealth index of the study participants. First, we selected variables incorporated from the questionnaire. The wealth index (WI) calculated using a household’s ownership of selected assets, such as hoe, and livestock, radio, television, cellphone, chair, table, bed, and refrigerator, main source of water supply, toilet, flooring, and exterior wall of the house, roofing materials, light source, and separate room for cooking. All variables were first dichotomized (1 = Yes, 0 = No) based on the ownership of each household asset. Principal component analysis (PCA) computed for constructing the WI of the study participants.The WI have compiled by assigning the household score and then dividing the distribution into five equal categories, from quintile one (lowest) to quintile five (highest), each having approximately 20% of the households. Bi-variable and multivariable binary logistic regression was used to identify associated factors of food cravings. The fitness of the model was checked using Hosmer- Lemeshow goodness of fit. Variables with a p-value <0.25 during the bi-variable logistic regression were further entered into the final model, multivariate logistic regression to control for potential confounding variables. Multi co-linearity was checked by (Variance inflation factor <10). Finally, the outputs of the bi-variable and multivariable binary logistic regressions were reported AOR together with a 95% Confidence interval.

### Ethical consideration

An ethical approval letter obtained from the Institutional Review Board (IRB) of Hawassa University College Medicine and Health (Ref.No.IRB/225/11). Likewise, an additional letter of support also secured from Sidama Regional Health Bureau. Verbal informed consent maintained from each study participant and this approved by the IRB. Information regarding any specific personal identifiers like the name of the mothers not collected and confidentiality of any personal information maintained throughout the study period.

## Result

### Socio-economic and demographic characteristics

Of the 586 pregnant women, 530 interviewed voluntarily to make a response rate of 90.4%. The majority, 421 (79.4%) of the respondents were rural residents. The median (IQR) age of the women was 26 (22, 30) years. Almost equal proportion of women, 253 (47.7%) and their husbands, 250 (47.2%) had completed primary education. The vast majorities of the study participant’s, 388 (73.2%) were housewive. Regarding the wealth quintile, the lowest and the middle were nearly equally represented with a lowest-to-middle ratio of 0.91 **(Table 1).**

**Table 1:** Socio-economic and demographic characteristics of the pregnant women Southern Ethiopia, 2020.

Variable (N = 530)	Frequency (N)	Percentage (%)
**Residence**		
Urban	109	20.6
Rural	421	79.4
**Maternal age in years**		
≤20	106	20
20–34	345	65.1
≥35	79	14.9
**Religion**		
Protestant	393	79.1
Orthodox	29	5.8
Muslim	67	13.1
**Educational status of women**		
No formal education	151	28.5
Primary	253	47.7
Secondary	66	12.5
Above secondary	60	11.3
**Educational status of husbands**		
No formal education	90	17.0
Primary	250	47.2
Secondary	111	20.9
Above secondary	79	14.9
**Occupation of the women**		
Housewife	388	73.2
Government employed	50	9.4
Merchant	58	10.9
Others*	34	6.4
**Occupation of husbands**		
Farmer	225	42.5
Merchant	192	36.2
Government employed	70	13.2
Others*	43	8.1
**Wealth quantile**		
Poor	207	39.1
Middle	97	18.3
Rich	226	42.6
**Family Size**		
≤ 2	340	64.2
3–4	130	24.5
≥5	60	11.3

*[Students, farmer, daily laborer]

### Pregnancy, meal pattern and anthropometric characteristics of the study participants

More than half, (52.6%) of the study participants were in their second trimester of pregnancy. The majorities, (65.3%) of the study participants have ANC follow-up during the current pregnancy. Of which, 70 (13.2%) of them were attended only for one time. Small proportion, (35.8%) of the study participants were grandparous. Out of the total study participants, 296 (55.6%) and 148 (27.9%) of the pregnant women experienced nausea and vomiting during their current pregnancy respectively. Out of the total study participants, 359 (67.7%) reported food aversion to at least one food during their current pregnancy. Nearly half, 254 (47.9%) of the study participants eat three times per day. Regarding the nutritional status of the pregnant women, the median (IQR) of MUAC measures of the study participants was 23 (20, 24) cm. Thirty six percent (194), of the study participants were found to be undernourished (MUAC < 23 cm) **(Table 2).**

**Table 2:** Pregnancy, meal pattern and anthrpometric characteristics of pregnant women Sidama Region, Southern Ethiopia 2021.

Variable (N = 530)		Frequency	Percentage (%)
Trimester of pregnancy	Second	279	52.6
	Third	251	47.4
ANC follow-up	Yes	346	65.3
	No	184	34.7
Number of ANC (n = 346)	1^st^	70	13.2
	2^nd^	193	36.4
	3^rd^	50	9.4
	4^th^	33	6.2
Number of pregnancies	≤ 2	340	64.2
	>2	190	35.8
Avoided any food in the current pregnancy	Yes	359	67.7
	No	171	32.3
**Number of meal per day**	Two times	110	20.8
	Three times	254	47.9
	≥Four times	166	31.3
**Skipped meal**	Yes	110	20.8
	No	420	79.2
**Type of meal skipped (n = 110)**	Lunch	75	68.2
	Dinner	35	31.8
**Eating additional meal**	Yes	166	31.3
	No	364	68.7
Pica practice	Yes	91	17.2
	No	439	82.8
Nausea	Yes	296	55.8
	No	234	44.2
Vomiting	Yes	148	27.9
	No	382	72.1
**MUAC**	< 23cm	194	36.6
	≥ 23cm	336	63.4

### Magnitude of food cravings among pregnant women

In this, study fifty-eight percent, 309 (58.3%) [95%, CI: 54.2%-62.8%] of the study participants reported food cravings during the current pregnancy. About, 113 (36.6%) of participants crave only one type of food. Meat (71.5%), Fruits (41.7%), and dairy products (22%) found to be the most frequent type of foods mentioned by the study participants. Regarding participants’ reasons for reported food cravings, more than three-in-seven (44.3%) of the study participants mentioned having a greater desire for a particular food. Merely, half of the food cravers, 158 (51.1%) were able to consume the food they were craved **(Table 3).**

**Table 3:** Magnitude and types of food craving among pregnant women in Sidama Regional State, Southern Ethiopia, 2021.

Variable (n = 530)	Frequency (N)	Percentage (%)
**Food craved**		
Yes	309	58.3
No	221	42.3
**Number of foods craved (n = 309)**		
1	113	36.6
≥2	196	63.4
**Types of foods craved**		
Cereal products	19	6.14
Roots and tubers	4	1.3
Legumes	4	1.3
Vegetables	40	12.9
Fruits	129	41.7
Meat	221	71.5
Egg	28	9.0
Fish	19	6.14
Dairy products	68	22.0
Kocho/enset	8	2.5
Coffee	1	0.3
Sweets/sugars	3	1
**Reasons for food craving**		
Greater desire to the food	134	43.3
Flavor and taste of the food	24	7.8
Don’t know	128	41.4
Believe a call/demand by the fetus	56	18.12
Prevent nausea and vomiting	6	2.0
**Able to consume craved food**		
Yes	158	51.14
No	151	48.86

### Factors associated with food craving

After controlling for the potential confounding variables by the multivariate analysis: Age of women: 20–34 years of age [AOR = 1.84(1.06–3.17)], government employed [AOR = 3.85 (1.64–9.04)], ANC [AOR = 0.60 (0.39–0.92)], Pica practice[AOR = 4.49 (2.35–8.56)], Lowest wealth quintile[AOR = 0.40 (0.20–0.83)], Skipping meal [AOR = 2.15 (1.26–3.68)] and Under-nutrition[AOR = 1.60 (1.02–2.51)] were identified to be statistically associated with food cravings.

Pregnant women aged 20–34 years were 84% [AOR = 1.84(1.06–3.17)] more likely to experience food cravings compared to pregnant women aged 35 and older years. Government employed pregnant women were 3.85 [AOR = 3.85 (1.64–9.04)] times more likely to experience food cravings as compared to pregnant Housewives. Moreover, pregnant women who had ANC visits were 40% [AOR = 0.60 (0.39–0.92)] less likely of experiencing food cravings as compared to women who have no ANC visits. Pregnant women who had practiced pica were nearly 4.5 [AOR = 4.49 (2.35–8.56)] times higher likelihood of experiencing food cravings as compared to their counterparts. Likewise, pregnant women who are in the lowest wealth quintile had 60% [AOR = 0.40 (0.20–0.83)] lower odds of food craving as compared to the women who are in the highest wealth quintile group. Pregnant women who skipped meals were 2 [AOR = 2.15 (1.26–3.68)] times more likely of experiencing food cravings as compared to the women who did not skip their meals. On the other hand, under-nourished pregnant women were 60% [AOR = 1.60 (1.02–2.51)] more likely of experiencing food cravings as compared to their counterparts (Table 4).

**Table 4:** Factors associated with food craving during pregnancy in Sidama Regional State, Southern Ethiopia, 2021.

Variables		Food cravings		COR (95% CI)	AOR (95% CI)
		Yes no (%)	No no (%)		
Age	<20	68 (22.0)	38 (17.2)	2.92 (1.59–5.34)	1.25 (0.52–3.02)
	20–34	211 (68.3)	134 (60.6)	2.57 (1.55–4.25)	1.84(1.06–3.17) *
	≥35	30 (9.7)	49 (22.2)	1	1
Residence	Urban	64 (20.7)	45 (20.4)	1.02 (0.66–1.56)	--
	Rural	245 (79.3)	176 (79.6)	1	--
Occupational status	Housewife	219(70.9)	169 (76.5)	1	1
	Government employed	42 (13.6)	8 (3.6)	4.05 (1.85–8.85)	3.85 (1.64–9.04)**
	Merchant	32 (10.4)	26 (11.8)	0.95(0.54–1.65)	1.08(0.55–1.84)
	Others#	16 (5.2)	18 (8.1)	0.68 (0.34–1.38)	0.67 (0.31–1.43)
Educational status	No formal education	85 (27.5)	66 (29.9)	1	---
	Primary	140 (45.3)	113 (51.1)	0.96 (0.64–1.44)	---
	Secondary	37 (12)	29 (13.1)	0.99(0.55–1.77)	---
	Teritiary+	47 (15.2)	13 (5.9)	2.80 (1.40–5.61)	---
Wealth quantile	Lowest	35 (41.7)	49 (58.3)	0.49 (0.27–0.89)	0.40 (0.20–0.83)*
	Second	75 (61)	48 (39)	1.09 (0.64–1.85)	0.88 (0.45–1.71)
	Middle	61 (62.9)	36 (37.1)	1.18 (0.67–2.08)	1.16 (0.61–2.20)
	Fourth	75 (63)	44 (37)	1.19 (0.69–2.03)	1.07 (0.59–1.97)
	Highest	63 (58.9)	44 (41.1)	1	-
ANC	Yes	187(55.8)	148(44.2)	0.76 (0.53–1.10)	0.60 (0.39–0.92)*
	No	100(61.7)	62(38.3)	1	1
Number of ANC	1^st^ visit	35 (18)	35 (23)	1	1
	2^nd^ visit	108 (55.7)	85 (55.9)	1.27 (0.73–2.19)	1.27 (0.73–2.19)
	3^rd^ visit	25 (12.9)	25 (16.4)	1.00 (0.48–2.06)	1.00 (0.48–2.06)
	4^th^ visit	26 (13.4)	7 (4.6)	3.71 (1.42–9.67)	3.71 (1.42–9.67)
Parity	Primiparous	99 (69.2)	44 (38.8)	1.89 (1.26–2.85)	1.70 (0.86–3.33)
	Multiparous	210 (54.3)	177 (45.7)	1	1
Pica practice	Yes	80 (86)	13 (14)	5.59 (3.02–10.34)	4.49 (2.35–8.56)***
	No	220 (50.1)	219 (49.9)	1	1
Nausea	Yes	179 (57.9)	117 (52.9)	1.24 (0.86–1.73)	--
	No	130 (42.1)	104 (47.1)	1	--
Vomiting	Yes	93(30.1)	55 (24.9)	1.29 (0.88–1.91)	
	No	216 (69.9)	166 (75.1)	1	
Food aversion	Yes	212 (68.6)	147 (66.5)	1.10 (0.76–1.59)	--
	No	97 (31.4)	74 (33.5)	1	--
Additional meal	Yes	103 (33.3)	63 (28.5)	1.25 (0.86–1.82)	
	No	206 (66.7)	158 (71.5)	1	-
Number of meal per day	Two	80 (25.9)	30 (13.6)	1.16 (0.96–2.75)	--
	Three	126 (40.8)	128 (57.9)	0.60 (0.40–0.89)	--
	Four and above	103 (33.3)	63 (28.5)	1	--
Skipping meal	Yes	80 (25.9)	30 (13.6)	2.22 (1.40–3.52)	2.15 (1.26–3.68)**
	No	229 (74.1)	191 (86.4)	1	1
Nutritional status	Under-nourished	123 (63.4)	71 (36.6)	1.39 (0.97–2.00)*	1.60 (1.02–2.51)*
	Well nourished	186 (55.5)	150 (44.5)	1	1

*** P-value < 0.001,

** P-value < 0.01,

* P-value < 0.05, COR: Crude Odd Ratio, AOR: Adjusted Odd Ratio

### Factors associated with nutritional status of pregnant women

After controlling for the potential confounding variables by multivariable binary logistic regression analysis, wealth quintile, ability to consume craved food, and food cravings found to be statistically significant factors associated with the nutritional status of pregnant women. Accordingly, pregnant women in the lowest and second wealth quintile had 19 [AOR = 19.81 (7.30–53.75)] and 18 [AOR = 18.57 (7.07–48.78)] times higher odds of being undernourished as compared to women in highest quintile groups respectively. Similarly, pregnant women in the third and fourth wealth quintile groups had 6.5 [AOR = 6.53 (2.17–17.25)] and 3.9 [AOR = 3.93 (1.55–9.95)] times higher odds of being undernourished as compared to women in highest wealth quintile group respectively. Likewise, pregnant women who had not experienced food cravings were 41% less likely of being under-nutrition as compared to pregnant women who had experienced food cravings during the current pregnancy [AOR = 0.59 (0.38–0.92)]. On the other hand, pregnant women who were unabled to consume the craved food had 2 times higher odds of being undernourished as compared to pregnant women who were abled to consume the craved food during the current pregnancy [AOR = 2.07, 95%CI (1.22–3.51)] **(Table 5).**

**Table 5:** Factors associated with nutritional status of pregnant women in Sidama Regional State, Southern Ethiopia, 2021.

Variables		Nutritional Status		COR (95% CI)	AOR (95% CI)
		Malnourished N (%)	Wellnourished N (%)		
Residence	Urban	22 (20.2)	87 (79.8)	0.36 (0.22–0.60)	1.03 (0.49–2.17)
	Rural	172 (40.9)	249 (59.1)	1	1
Number of meal per day	Two	66 (60)	44 (40)	1	1
	Three	84 (33.1)	170 (66.9)	9.75(2.09–45.33)	0.62 (0.36–1.07)
	Four	42 (27.8)	109 (72.2)	3.21(0.70–14.56)	0.72 (0.39–1.32)
	Five	2 (13.3)	13 (86.7)	2.50(0.54–11.57)	0.85 (0.15–4.71)
Wealth quintile	Lowest	50 (59.5)	34 (40.5)	21.00 (8.70–50.72)	19.81(7.30–53.75)***
	Second	76 (61.8)	47 (38.2)	23.10 (9.89–53.95)	18.57(7.07–48.78)***
	Middle	32 (33)	65 (67)	7.03 (2.93–16.87)	6.53 (2.17–17.25)***
	Fourth	29 (24.4)	90 (75.6)	4.60 (1.92–11.02)	3.93 (1.55–9.95)**
	Highest	7 (6.5)	100 (93.5)	1	1
Food craving	No	71 (31.1)	150 (68.9)	0.71 (0.49–1.02)	0.59 (0.38–0.92)*
	Yes	123 (39.8)	186 (60.2)	1	1
Able consume craved food	Yes	45 (28.5)	113 (71.5)	1	1
	No	149 (40.1)	223 (59.9)	1.59 (1.06–2.37)	2.07 (1.22–3.51)***
Additional meal	Yes	44 (26.5)	122 (73.5)	1	1
	No	150 (41.2)	214 (58.8)	1.94 (1.29–2.90)	0.94 (0.57–1.54)
Skipping meal	Yes	66 (60)	44 (40)	1	1
	No	128 (30.5)	292 (69.5)	0.29(0.18–0.45)	0.61(0.36–1.02)

*** P-value < 0.001,

** P-value < 0.01,

* P-value < 0.05, Crude Odd Ratio, AOR: Adjusted Odd Ratio

## Discussion

This study aimed to assess the magnitude of foods craving, types of food craved and averted, and factors affecting food cravings, and to measure the effect of food cravings on the nutritional status of pregnant women during pregnancy in Sidama Regional State, Southern Ethiopia, 2021. According to the study result, the prevalence of food cravings found in this study was 58.3% [95%, CI: 54.2%-62.8%]. Similarly, around thirty-six percent, 194 of the study participants found to be undernourished (MUAC < 23 cm). Age of women (20–34 years), the government employed, ANC, Pica practice, lowest wealth quintile, and skipping meals identified to be positively associated with food cravings. Whereas, wealth quintile and ability to consume craved food found to be statistically significant factors associated with the nutritional status of pregnant women. Moreover, our study result found that maternal undernutrition and food cravings were statistically associated.

The global prevalence of food cravings varies from 38 to 79%, being less frequent in European populations and more common in the African continent [3, 4]. The prevalence of food cravings found in this study, 58.3% [95%, CI: 54.2%-62.8%] is supportive of the above evidence. A similar magnitude was also reported by other studies [10, 13]. However, it is slightly lower than the findings of previous similar studies done in Nigeria (74.8%) [14], Ghana (67.7%) [15], Hadiya, Ethiopia (71%) [9], and two other studies in Tanzania (79%) and (73.5%) [8, 16]. It is acknowledged that pregnant women in low-income countries crave mainly livestock origin such as meat, yogurt, cheese and milk followed to a lesser extent by vegetables, fruits, and grains [10]. The present study results are relatively consonant with the above evidence, as the most desired foods were meat (71.1%), fruits (41.5%), and dairy products (22.3%). whereas vegetables (13%) and cereal products (6%) were found to be craved to a lesser extent by the study participants. This is inconsonant with the studies’ reports [6, 8, 9].

The present study found that governmental employed pregnant women were more likely of experiencing food cravings as compared to housewive. This finding is consonant with a study reported in Ghana [15]. This could be due to the reason that an employed woman would have good nutritional knowledge and they crave different foods that could help their own and their baby’s health. Moreover, research evidence also suggested that women exposed to nutrition information were more likely to have good dietary practices than women have not exposed to nutrition information [17]. On the other hand, our study result found that pregnant women who are in the lowest wealth quintile had lower odds of food cravings as compared to the women who are in the highest wealth quintile. A similar result found in another study [9]. This might be the reason that poor women might have fewer food items choices for craving as commonly as women who have more choices.

On the other hand, pregnant women who are found in the young age group were more likely to experience food cravings than pregnant women aged > 35 years. Consistent with our study result, different studies reported that younger age was associated with a greater number of cravings [15, 18]. This might be because as the age of the woman increases her experience with the physiology of pregnancy also increased. As a result, she could resist or overcome this change with predetermined experiences or skills she got. In the present study, meal skippers found to be 2 times more likely to experience cravings than those who did not skip a meal. This could be becasue of these women suffer from a shortage of essential nutrients due to skipping a meal and need to compensate for the deficiency they encounter due to their practice by craving nutritious foods. Poor dietary practice could result in a broad range of short and long-term negative consequences on both the mother’s and her baby’s health and nutritional status. The impact of poor dietary habits and feeding practices of women do not end up with deprived birth outcome and nutritional status of the baby however, its effect may also influence children’s health during adulthood [19].

Pregnant women who had practiced pica have a higher likelihood of experiencing food cravings as compared to their counterparts. Consistent with our study result different studies revealed that craving for nonfood items reduces the intake of nutritious foods which in turn leads to inadequate dietary intake of essential nutrients [8, 20, 21].On the other hand, our study result found that pregnant women who skipped meals were more likely to experience food cravings than women who did not skip meals. Skipping meals during pregnancy can affect the blood glucose level and also increase cravings for junk foods, which have empty calories to meet the nutritional demand of the pregnant mother [22]. This might be because a mother who skips meals might have a higher affinity for searching for foods to satisfy their nutritional demands.

In this study three-in-eight, 36.6% of the study participants found to be undernourished. This is higher than the 2016 national estimates of 22% [23], a study conducted in Gondar town, Northwest Ethiopia, 14.4% [24], and Dessie town, northeastern Ethiopia, 19.5% [25]. Similarly, one in three (30.2%) pregnant women ate food more than three times per day. This is relatively consistent with the study report from Ghana 37.7% [15]. However, lower compared to the finding from Limpopo Province, South Africa, in which 46% of the study participants ate more than three times per day [26]. The observed difference in meal patterns of the pregnant women might be due to varied socio-economic characteristics, and geographical and methodological differences, where this study relatively had a large calculated sample size. Regarding meal skipping during the current pregnancy, (20.5%) of the women skipped at least one meal per day. This is by far lower than the study report from Northern Ethiopia, 62% [17]. Nearly seven in ten, 364 (68.8%) of the study participants did not eat an additional meal during the current pregnancy. On the contrary, a study report from Northern Ethiopia showed that 71% of the women had additional meals during their recent pregnancy [17]. The discrepancy might be attributable to differences in sample size, geographical location, and socio-economic and cultural conditions of the study populations.

The study result revealed that the odds of undernutrition have decreased as the wealth quintile of pregnant women increases. Similarly, the odds of undernutrition were higher among pregnant who are unable to consume the food which they craved for it. Similar studies conducted in Southern Ethiopia showed that those craving women who got the desired foods had significantly higher weight gain [6, 9]. Likewise, prior studies results found that most women increased their food consumption during pregnancy, because of the availability of sufficient food in the household [23, 27, 28]. Our study result also showed that merely 48.9% of pregnant women were able to get the food that they craved for it. This might be because pregnant women with the lowest wealth quintiles may not able to consume diverse and healthy diets with essential micronutrients due to their limited purchasing power.

In the present study, the nutritional status of pregnant women and food cravings are statistically associated. Meaning that pregnant women who had not experienced food cravings were less likely of being undernourished compared to pregnant women who had experienced food cravings. This finding supports the evidence, which stated that specific food cravings might be a response to a nutritional deficiency [10]. Similarly, our study results corroborate the evidence that pregnant women who are not eating a balanced diet during their pregnancy have a higher risk of craving junk foods [22]. A study result reported that mothers who eat junk food during pregnancy are more likely to have nutritional problems [29]. On the other hand, a prior study found that a craving for pica is statistically associated with the undernutrition of pregnant mothers [6]. Meanwhile, our study found that more than one-in-six in the pregnant women craved non-food items (pica). This indicates that the majority of women who craved foods are not the needed ones to meet the nutritional demand of the pregnant mother.

Additionally, it has often been posited that food cravings are key drivers of food choice and met with the consumption of the craved food during pregnancy [10, 28], but in this study merely half of the food cravers were able to consume the food they craved for it. This implies that the magnitude of undernutrition was higher among pregnant women who were unable to consume the food they craved for it. On the other hand, as our study revealed, a large number of the study participants skipped their meals during their current pregnancy. Unfortunately, skipping meals during pregnancy is associated with increased cravings for junk foods [22] and has a considerably lower chance of having adequate nutrition due to the reduced meal frequency [30]. However, different studies found no significant difference in nutritional status among pregnant women with cravings and without cravings for specified food [4, 9, 13, 15]. The observed differences might be due to the differences in sample size and socio-demographic characteristics of the study populations.

### Strength and limitation of the study

Being a community-based study and having a relatively a larger sample size were among the strength of the study. On the other hand, the limitation of the study could be a recall bias, relying only on anthropometric measurement (MUAC) to determine the nutritional status of pregnant women, and fail to determine the temporal relationship between food cravings and the nutritional status of women.

## Conclusion

The prevalence of food cravings found in this study is comparable to the global levels. However, the magnitude of under nutrition among pregnant women found to be comparably higher than the global figures. The study identifies that age of women, government employed, ANC, pica practice, lowest wealth quintile, skipping meal, and under-nutrition identified to factors associated with food cravings. On the other hand, wealth quintile, ability to consume craved food, and food cravings found to be statistically significant factors associated with the nutritional status of pregnant women. Moreover, our study result found that maternal under-nutrition and food cravings were statistically associated. A large number of the study participants skipped their meals during their current pregnancy, which implies that, the dietary patterns of the women are suboptimal. Since food cravings linked to nutritional status of the pregnant woman, an emphasis has given to this physiological change to address the maternal nutrition status. Health care professionals have to strengthen counseling and follow up of pregnant women to have a healthy diet and to avoid craving for junky foods during pregnancy, which promotes healthy pregnancy outcomes. Further, we recommend prospective longitudinal research on this area to determine the temporal relationship between food craving and the nutritional status of pregnant women.

## Supporting information

S1 File English version questionnaire. (PDF)
