# Supplementary material for: Magnitude and effects of food cravings on nutritional status of pregnant women in Southern Ethiopia: A community-based cross sectional study
Source: PLoS One. 2022 Oct 13;17(10):e0276079. doi: 10.1371/journal.pone.0276079 (PMC9560518; doi:10.1371/journal.pone.0276079)
Supplement: S1 File — (PDF) [file pone.0276079.s001.pdf]

English version information sheet and consent form

Hello, my name _______________. I am working as a data collector for the study conducted by group of researchers from Hawassa University School of Nutrition, Food Science and Technology, Department of Applied Human Nutrition. The purpose of this study is to assess prevalence of food cravings and aversions during pregnancy and association with nutritional status of pregnant women in your area. I would like to ask some questions about your socio – demographic economic characteristics, meal pattern, and your MUAC will be measured. The result of study will be important to pregnant women, depending on the result of study the government or other concerned body which will act in a way to correct the pregnant women nutrition and related health services, if there is any benefit, you will be one of those who will get this benefit. There will be no payment for this particular study. There is no risk or discomfort you should fear as a result of participating in this study. You do not need to provide your name. Please, whatever information you provide will be kept strictly confidential and information identifying you will never be released to anyone outside of this information collection activity. I expect the interview may take about 20 – 30 minutes.

Participation in this study is voluntary, and you can choose not to answer any individual question or all of the questions. You may stop the interview completely at any time you wish without any consequences at all. However, we hope that you will participate in this study and provide the correct information to all questions.

Is all the information given above is clean to you and so can I proceed 1. YES 2. No

Yes proceed_______________

Name of data collector______________ sign_________ date__________

Name of supervisor___________________ sign___________ date______________

Address of Contact person: Mobile Phone: (+ 251977212376)

Email address: abelyalew@gmail.com

English version questionnaire for data collection

Questionnaire code ________________

Name of the kebele ______________________

Name of the data collector __________________

Date ______________________

Section 1– SOCIO DEMOGRAPHIC DATA				
S.No	Question	Answer	Skip	Remark
101	Age of the respondent in completed years	---------- years		
102	Residence	Urban<br>Rural		
103	Religion	Protestant<br>Orthodox<br>Muslim<br>Other specify…………		
104	Ethnicity	Sidama<br>Amhara<br>Oromo<br>Wolayita<br>Other specify ……		
105	Marital status	Single<br>Married<br>Divorced<br>widowed		
106	Level of education	No formal education<br>Primary		

		Secondary<br>Above secondary		
107	Level of education of the husband	No formal education<br>Primary<br>Secondary<br>Above secondary		
108	Occupation	House wife<br>Daily laborer<br>Student<br>Government employed<br>Merchant<br>Farmer<br>Other specify……….		
109	Occupation of the husband	Daily laborer<br>Student<br>Government employed<br>Merchant<br>Farmer<br>Other specify………		
110	How many children do you have?	------------------------		
111	Which months of pregnancy are you in?	-------------------------		
112	Do you have had ANC follow up?	Yes<br>No	If no skip to Q No. 114	
113	If yes how many times?	--------------------------		
114	Do you suffer from nausea and vomiting during your pregnancy?	Yes only nausea<br>Yes only vomiting<br>Yes both nausea and vomiting<br>No		

SECTION 2 – ECONOMIC DATA						
S.No	Question	Answer			Skip	Remark
200	Does any member of your household own agricultural land?	Yes No			If no skip to Q.No. 202	
201	If you say yes, how many (local units) of agricultural land do members of your house hold own?	------------------------				
202	Does your house hold own any livestock, herds, other farm animals or poultry?	Yes No			If no skip to Q No. 204	
203	If you say yes, please specify which of the following animals do in your house hold own?	Milk cows	1.Yes	0.No		
		Oxen/Bulls	1.Yes	0.No		
		Horses	1.Yes	0.No		
		Donkeys/ mules	1.Yes	0.No		
		Goats	1.Yes	0.No		
		Sheep	1.Yes	0.No		
		Chickens	1.Yes	0.No		
204	Does your house hold have	Electricity	1.Yes	0.No		
		Radio	1.Yes	0.No		
		Television	1.Yes	0.No		
		Mobile	1.Yes	0.No		
		Cellphone	1.Yes	0.No		
		Refrigerator	1.Yes	0.No		
		Table	1.Yes	0.No		
		Chair	1.Yes	0.No		
		Bed with cotton/spon	1.Yes	0.No		

		ge/spring material?				
205	What type of fuel does your house hold usually use for cooking?	Electricity<br><br>Natural gas<br><br>Biogas<br><br>Kerosene<br><br>Charcoal<br><br>Wood<br><br>Straw/shrubs/grass<br><br>Agricultural crops<br><br>Animal dung<br><br>No food cooked in house hold<br><br>Other specify…………….				
206	Do you have separate room for cooking?	Yes No				
207	What is the main material of the floor of your house?	Natural floor<br>earth/sand<br>wood<br>cement/ceramic tiles<br>other specify				
208	What is the main material of the roof of your house?	Iron/aluminum steel<br>Grass<br>Plastic				

		Other specify		
209	What is the main material of exterior walls of your house	Leaf/grass<br>Wood and mud<br>Plastic sheets<br>Card board<br>Wood<br>Other specify ………………		
210	What is your households' main source of water?	Tap water<br>Well<br>River<br>Spring<br>Other specify ……		
211	Where is that water source located?	In own dwelling<br>In own yard /plot<br>Elsewhere		
212	Does your household have private latrine?	Yes<br>No	If no skip to Q No. 300	
213	If yes, what is the type of your household latrine?	Flush or pour flush latrine<br>Ventilated improved pit latrine<br>Pit latrine with slab<br>Pit latrine without slab/open pit<br>Other specify…………		
214	Do you share this toilet facility with other households?	Yes<br>No		
215	If yes with how many households do you share your toilet?	-------------------------		

SECTION 3 – DIETARY BEHAVIOR DATA				
S.No	Question	Answer	Skip	Remark
300	How many times you eat food per day?	------------------------		
301	Do you skip meals?	Yes<br>No	If no skip to Q No. 303	
302	If yes which meal do you skip?	Breakfast<br>Lunch<br>Dinner		
303	Do you eat additional meal?	Yes<br>No	If no skip to Q No. 305	
304	If yes how many times in a day?	-------------------------		
305	Did you experience a strong dislike for a certain food (aversions) during current pregnancy?	Yes<br>No	If no skip to Q No. 308	
306	If yes, please name the foods you avoid.	----------------------------<br>---------------------------		
307	What was your reason?	----------------------------<br>---------------------------		
308	Did you experience a strong and sudden desire for any food (food craving)during your pregnancy?	Yes<br>No	If no skip to Q.No. 313	
309	If yes please name the foods you desired strongly.	----------------------------<br>----------------------------		

310	What was your reason for strong desire?	______________________ ----------------------------		
311	Do you get and consume all the foods you desired strongly?	Yes<br>No	If yes skip to Q.No. 313	
312	What do you do if you don't get the food you craved?	----------------------------<br>----------------------------		

Section 4 – ANTHROPOMETRIC STATUS DATA

Mid-Upper Arm Circumference (MUAC)

Measurement 1 ________________cm

Measurement 2 ________________cm

Average _________________cm

THANK YOU FOR YOUR PATICIPATION!!
